# Correction: Narongkiatikhun et al. Immunogenicity and Safety of Homologous and Heterologous Prime-Boost of CoronaVac^®^ and ChAdOx1 nCoV-19 among Hemodialysis Patients: An Observational Prospective Cohort Study. *Vaccines* 2023, *11*, 175

**DOI:** 10.3390/vaccines11101538

**Published:** 2023-09-28

**Authors:** Phoom Narongkiatikhun, Kajohnsak Noppakun, Romanee Chaiwarith, Poramed Winichakoon, Surachet Vongsanim, Yuttitham Suteeka, Karn Pongsuwan, Prit Kusirisin, Nuttanun Wongsarikan, Kanda Fanhchaksai, Chantana Khamwan, Dararat Dankai, Vuddhidej Ophascharoensuk

**Affiliations:** 1 Division of Nephrology, Department of Internal Medicine, Faculty of Medicine, Chiang Mai University, Chiang Mai 50200, Thailand; phoom.n@cmu.ac.th (P.N.); kajohnsak.noppakun@cmu.ac.th (K.N.); surachet.w@cmu.ac.th (S.V.); yuttitham.s@cmu.ac.th (Y.S.); karn.p@cmu.ac.th (K.P.); prit.kusirisin@cmu.ac.th (P.K.); 2 Division of Infectious Diseases and Tropical Medicine, Department of Internal Medicine, Faculty of Medicine, Chiang Mai University, Chiang Mai 50200, Thailand; romanee.c@cmu.ac.th (R.C.); poramed.wi@cmu.ac.th (P.W.); 3 Department of Internal Medicine, Faculty of Medicine, Chiang Mai University, Chiang Mai 50200, Thailand; nuttanun.w@cmu.ac.th; 4 Division of Hematology and Oncology, Department of Pediatrics, Faculty of Medicine, Chiang Mai University, Chiang Mai 50200, Thailand; kanda.f@cmu.ac.th; 5 Immunology Laboratory, Diagnostic Laboratory, Faculty of Medicine, Chiang Mai University, Chiang Mai 50200, Thailand; chantana.k@cmu.ac.th (C.K.); dararat.d@cmu.ac.th (D.D.)

The authors wish to make the following correction to this paper [1]:

In the original publication, in “*Section 2.1. Study Design and Participants*”, there was a mistake in the initial date of patient recruitment, which should be 4 June 2021, instead of 1 June 2021. The corrected date appears below:

“We conducted a single-center, observational prospective cohort study involving MHD patients between 4 June and 31 December 2021 at Maharaj Nakorn Chiang Mai Hospital, which is an affiliated hospital of Chiang Mai University, Thailand.”

The authors state that the scientific conclusions are unaffected. This correction was approved by the Academic Editor. The original publication has also been updated.
