# Supplementary material for: Immunogenicity and Safety of Homologous and Heterologous Prime-Boost of CoronaVac® and ChAdOx1 nCoV-19 among Hemodialysis Patients: An Observational Prospective Cohort Study
Source: Vaccines (Basel). 2023 Mar 23;11(4):715. doi: 10.3390/vaccines11040715 (PMC10146055; doi:10.3390/vaccines11040715)
Supplement: Supplementary file 1 [file vaccines-11-00715-s001.zip › vaccines-2243165-supplementary.pdf]

Table Legends

Table S1. Magnitude of anti-spike RBD IgG and sVNT at specific time point for each vaccine regimen.

Table S2. Comparison rate of the positive result of RBD-specific antibody and the seroconversion rate between each vaccine regimen.

Table S3. Full univariate analysis of factors associated with the seroconversion measured by sVNT among maintenance hemodialysis patients.

Table S1. Magnitude of anti-spike RBD IgG and sVNT at specific time point of each vaccine regimen.

Immunogenicity	SV+SV (N=16)			SV+AZ (N=25)			AZ+AZ (N=89)				P-value*	P-value†
	Visit 1	Visit 2	Visit 3	Visit 1	Visit 2	Visit 3	Visit 1	Visit 2	Visit 3	Visit 4		
**Anti-spike RBD IgG**												
Negative	0<br>(0, 4.90)	2.25<br>(0.26, 37.97)	24.62<br>(16.47, 32.76)	0<br>(0, 9.71)	1.50<br>(0, 19.66)	16.92<br>(9.58, 28.59)	0<br>(0, 14.73)	6.16<br>(0, 44.07)	8.72<br>(0, 47.42)	21.72<br>(2.26, 49.46)	0.872	0.816
	16/16 (100.0)	16/16 (100.0)	2/16 (12.5)	25/25 (100.0)	24/25 (96.0)	3/25 (12.0)	89/89 (100.0)	68/86 (79.1)	80/89 (89.9)	16/89 (18.0)	-	
Positive	-	-	139.34<br>(50.97, 895.10)	-	99.80<br>(99.80, 99.80)	460.95<br>(70.74, 3626.44)	-	88.07<br>(52.64, 456.61)	77.88<br>(55.80, 804.80)	208.85<br>(50.19, 1255.44)	<0.001	
	0/16 (0)	0/16 (0)	14/16 (87.50)	0/25 (0)	1/25 (4.0)	22/25 (88.0)	0/86 (0)	18/86 (20.9)	9/86 (10.1)	73/86 (82.0)	-	
**Neutralizing Ab**												
Negative	-10.04<br>(-17.25, -5.08)	-5.84<br>(-19.54, 5.67)	-6.18<br>(-11.02, -1.34)	-9.90<br>(-16.83, -0.46)	-7.67<br>(-12.31, 3.44)	-2.06<br>(-8.25, 12.72)	-7.63<br>(-17.54, 1.7)	-2.48<br>(-15.43, 15.49)	-0.43<br>(-16.6, 19.77)	4.33<br>(-13.57, 16.56)	0.390	0.289
	16/16 (100.0)	16/16 (100.0)	2/16 (12.5)	25/25 (100.0)	23/25 (92.0)	3/25 (12.0)	89/89 (100.0)	65/86 (75.6)	74/89 (83.2)	12/89 (13.5)	-	
Borderline	-	-	33.63<br>(20.82, 34.92)	-	-	-	-	28.42<br>(22.61, 34.26)	24.71<br>(20.48, 31.27)	27.25<br>(22.03, 33.22)	0.511	
	0/16 (0)	0/16 (0)	3/16 (18.8)	0/25 (0)	0/25 (0)	0/25 (0)	0/89 (0)	12/86 (14.0)	9/89 (10.1)	7/89 (7.9)	-	
Positive	-	-	59.06<br>(35.05, 97.30)	-	57.71<br>(52.48, 62.94)	92.60<br>(36.65, 99.18)	-	58.74<br>(37.42, 98.7)	45.40<br>(35.56, 91.63)	79.71<br>(35.05, 99.27)	0.080	
	0/16 (0)	0/16 (0)	11/16 (68.8)	0/25 (0)	2/25 (8.0)	22/25 (88.0)	0/89 (0)	9/86 (10.5)	6/89 (6.8)	70/89 (78.7)	-	

* Comparison magnitude of anti-spike RBD IgG and sVNT on day 28 after the second dose between each vaccine regimen

† Comparison rate of positive or seroconversion on day 28 after the second dose between each vaccine regimen

Table S2. Comparison rate of the positive result of RBD-specific antibody and the seroconversion rate between each vaccine regimen.

Antibody	SV+SV (N=16)	SV+AZ (N=25)	p-value	SV+SV (N=16)	AZ+AZ (N=89)	p-value	SV+AZ (N=25)	AZ+AZ (N=89)	p-value
RBD			1.000			0.733			0.762
Negative	2 (12.50)	3 (12.00)		2 (12.50)	16 (17.98)		3 (12.00)	16 (17.98)	
Positive	14 (87.50)	22 (88.00)		14 (87.50)	73 (82.02)		22 (88.00)	73 (82.02)	
Neutralize			0.100			0.399			0.501
Negative	3 (12.00)	2 (12.50)		2 (12.50)	12 (13.48)		3 (12.00)	12 (13.48)	
Borderline	0	3 (18.75)		3 (18.75)	7 (7.87)		0	7(7.87)	
Positive	22 (88.00)	11 (68.75)		11 (68.75)	70 (78.65)		22 (88.00)	70 (78.65)	

AZ-AZ, homologous AZD1222 regimen; SV-AZ, heterologous Sinovac-AZD1222 regimen; SV-SV, homologous Sinovac regimen

Table S3. Full univariate analysis of factors associated with the seroconversion measured by sVNT among maintenance hemodialysis patients.

Variable	Positive	Univariate Analysis		
		OR	95%CI	value
No.	103/130 (79.2)			
Age - (every 5-year increment in age)	63.2±14.4	0.81	0.66 - 0.99	0.040
Female	48/59 (81.4)	1.27	0.54 - 3.00	0.587
Vaccine regimen				
SV-AZ vs SV-SV		3.33	1.49 - 7.43	0.003
AZ-AZ vs SV-SV		1.67	0.93 - 3.01	0.085
SV-AZ vs AZ-AZ		1.99	1.03 - 3.83	0.039
Body mass index – kg/m^2	23.8±4.4	0.95	0.88 - 1.02	0.149
Comorbid disease				
Hypertension	98/124 (79.0)	0.75	0.08 - 6.74	0.800
Dyslipidemia	38/53 (71.7)	0.47	0.20 - 1.10	0.083
Diabetes mellitus	42/57 (73.7)	0.55	0.23 - 1.29	0.172

Variable	Positive	Univariate Analysis		
		OR	95%CI	value
Cardiovascular disease (MI, HF)	19/25 (76.0)	0.79	0.28 - 2.23	0.658
Cerebrovascular disease	3/4 (75.0)	0.78	0.08 - 7.81	0.833
Chronic obstructive pulmonary disease	2/2 (100.0)	-		
Connective tissue disease	6/8 (75.0)	0.77	0.15 - 4.06	0.761
Liver disease	5/6 (83.3)	1.33	0.15 - 11.85	0.800
Dialysis vintage – months	44 (27, 70)	1.00	0.99 - 1.01	0.696
Dialysis schedule				
1-2 T/week	19/25 (76.0)	Ref.		
3-4 T/week	84/105 (80.0)	1.26	0.45 - 3.56	0.658
Mode of hemodialysis				
Conventional hemodialysis	93/119 (78.2)	Ref.		
Online hemodiafiltration	10/11 (90.9)	2.80	0.34 - 22.86	0.338
Urine output - ml				
<200	23/28 (82.1)	Ref.		

Variable	Positive	Univariate Analysis		
		OR	95%CI	value
≥200	43/55 (78.2)	0.78	0.24 - 2.48	0.673
Iron supplement	65/82 (79.3)	1.01	0.42 - 2.42	0.989
Laboratory result				
Hemoglobin - g/dL	10.38±1.44	1.12	0.83 - 1.51	0.461
Hemoglobin <10	37/49 (75.5)	Ref.		
Hemoglobin ≥10	66/81 (81.5)	1.43	0.60 - 3.37	0.417
White blood cell - cells/mm^3	6654.0±2077.6	1.00	1.00 - 1.00	0.385
Polymorphonuclear - %	63.8±8.7	1.00	0.96 - 1.05	0.993
Lymphocyte - %	22.7±6.1	1.08	1.01 - 1.17	0.031
≤16	11 (10.7)	Ref.		
>16	92 (89.3)	4.92	1.81 - 13.38	0.002
Total Lymphocyte	1462.8±480.1	1.00	1.00 - 1.00	0.210
Platelet - x10^3 cells/mm^3	218 (170, 258)	1.00	1.00 - 1.00	0.186
Transferrin saturation - %	31 (24, 43)	1.01	0.98 - 1.03	0.567

Variable	Positive	Univariate Analysis		
		OR	95%CI	value
Ferritin - µg/L	460 (302, 724)	1.00	1.00 - 1.00	0.796
C-reactive protein - mg/L	3.12 (3.12, 7.7)	1.07	0.95 - 1.17	0.290
Albumin - g/dL	4 (3.9, 4.2)	1.91	0.57 - 6.37	0.292
Kt/v	1.6±0.5	1.94	0.73 - 5.15	0.182
Inadequate	32/43 (74.4)	Ref.		
Adequate*	71/87 (81.6)	1.53	0.64 - 3.65	0.344

All values are presented in number (%), unless otherwise specified.

* Defines by Kt/v ≥1.2 for 3 T/week of hemodialysis, and ≥2.1 for 2T/week of hemodialysis

AZ-AZ, homologous AZD1222 regimen; HF, heart failure; MI, myocardial infarction; SV-AZ, heterologous Sinovac-AZD1222 regimen; SV-SV, homologous Sinovac regimen
